# Effect of Chronic Psychological Stress on Liver Metastasis of Colon Cancer in Mice

**DOI:** 10.1371/journal.pone.0139978

**Published:** 2015-10-07

**Authors:** Lu Zhao, Jianhua Xu, Fang Liang, Ao Li, Yong Zhang, Jue Sun

**Affiliations:** 1 Department of Oncology, Putuo Hospital, Shanghai University of Traditional Chinese Medicine, Shanghai, China; 2 Interventional Cancer Institute of Chinese Integrative Medicine, Putuo Hospital, Shanghai University of Traditional Chinese Medicine, Shanghai, China; University of Heidelberg, GERMANY

## Abstract

Metastasis to the liver is a main factor in colorectal cancer mortality. Previous studies suggest that chronic psychological stress is important in cancer progression, but its effect on liver metastasis has not been investigated. To address this, we established a liver metastasis model in BALB/c nude mice to investigate the role of chronic stress in liver metastasis. Our data suggest that chronic stress elevates catecholamine levels and promotes liver metastasis. Chronic stress was also associated with increased tumor associated macrophages infiltration into the primary tumor and increased the expression of metastatic genes. Interestingly, β-blocker treatment reversed the effects of chronic stress on liver metastasis. Our results suggest the β-adrenergic signaling pathway is involved in regulating colorectal cancer progression and liver metastasis. Additionally, we submit that adjunctive therapy with a β-blocker may complement existing colorectal cancer therapies.

## Introduction

The overall incidence and mortality of colorectal cancer (CRC) have declined due to early diagnosis and new therapeutic methods [1]. However, liver metastasis still remains a significant challenge in prolonging survival. About 50~60% of patients develop liver metastasis, which has poor expected outcomes. Additionally, current therapeutic strategies are not effective [2]. Surgical resection is the only potential curative treatment for CRC liver metastasis. However, it is limited by indication and contraindication, and only 10~20% of patients with liver metastasis receive surgical treatment. The prognosis for patients with untreated liver metastasis is poor, and the expected 1-year survival rate is less than 30% [3–4]. To reduce morbidity and improve prognosis, it is essential to develop new therapeutics. Interestingly, epidemiological studies suggest that treatment with propranolol (a non-selective β-blocker) reduces the risks of some cancers, including CRC [5–8].

Recent studies suggest that a distressed emotional state is a potential risk factor for cancer progression. As cancer diagnosis can cause major distress, patients often experience fear, anxiety, and depression [9]. These signals can activate stress pathways, including the hypothalamic-pituitary-adrenal (HPA) axis and sympathetic nervous system (SNS), and elicit physiological responses. These pathways may produce neurotransmitters and hormones that alter the tumor microenvironment.

Catecholamine hormones, including norepinephrine (NE) and epinephrine (E), are elevated during stress, and play the key role in the stress response. These hormones activate receptors on tumor cells and regulate a wide variety of biological functions involved in cancer progression, including cellular proliferation, migration, invasion, and angiogenesis [10–12]. The activation of the β_2_-AR signaling pathway induced by catecholamine hormones plays a key role in cancer progression [13, 14].

Previous studies suggest that stress hormones-induced CRC cell proliferation is adrenoreceptor dependent and that chronic stress could promote the tumor growth of subcutaneously implanted colon carcinoma cells in a nude mice model through the β-AR signaling pathway [15, 16]. We hypothesized that chronic stress would promote CRC metastasis to the liver. Here we investigate how chronic stress affects liver metastasis using an *in vivo* metastatic model of colon cancer, and determine whether the β-AR signaling pathway is required for the progression of CRC.

## Materials and Methods

### Cell culture and animals

Human colon cancer cell line, HT–29, was purchased from the Type Culture Collection of the Chinese Academy of Sciences. HT–29 was cultured in McCoy’5A medium (Invitrogen), supplemented with 10% fetal bovine serum (Invitrogen), 100 U/ml penicillin, and 100 μg/ml streptomycin (Invitrogen). Cells were maintained at 37°C in a humidified atmosphere of 5% CO_2_.

Four-week-old male BALB/C nu/nu mice were purchased from Shanghai SLAC Experimental Animal Co., Ltd (license No: SCXK [Hu] 2012–0002) and maintained under SPF condition. All procedures performed were approved by the Animal Experimentation Ethics Committee of Putuo Hospital affiliated to Shanghai University of Traditional Chinese Medicine.

### Liver metastasis model

Mice were injected with HT–29 colon cancer cells in the spleen to produce liver metastasis. The mice were anesthetized with 2.5% pentobarbital sodium by peritoneal injection. After sterilization of the skin in the area of surgery, an abdominal incision paralleling the left subcostal margin was made. Tumor cells (1×10^7^) in 100 μl phosphate-buffered saline solution were injected into the spleen using a 29-G needle. After pressing the pinhole 1–2 min with aseptic cotton buds to prevent the cells from leaking out, spleen was returned into the peritoneal cavity. The wound was closed with 5–0 vicryl sutures.

### Chronic stress and drug administration protocol

Mice were randomly assigned into 6 groups: Blank-Control (BC, n = 10), Blank-Stress (BS, n = 10), Propranolol-Control (PC, n = 10), Propranolol-Stress (PS, n = 10), ICI118,551-Control (IC, n = 10) and ICI118,551-Stress (IS, n = 10). Mice in the stress groups experienced physical restraint for 6 hours per day for 35 days commencing 7 days before tumor cell injection. All the mice were sacrificed 28 days after tumor cell injection, and the spleen and liver were harvested and weighed.

**Fig 1:**
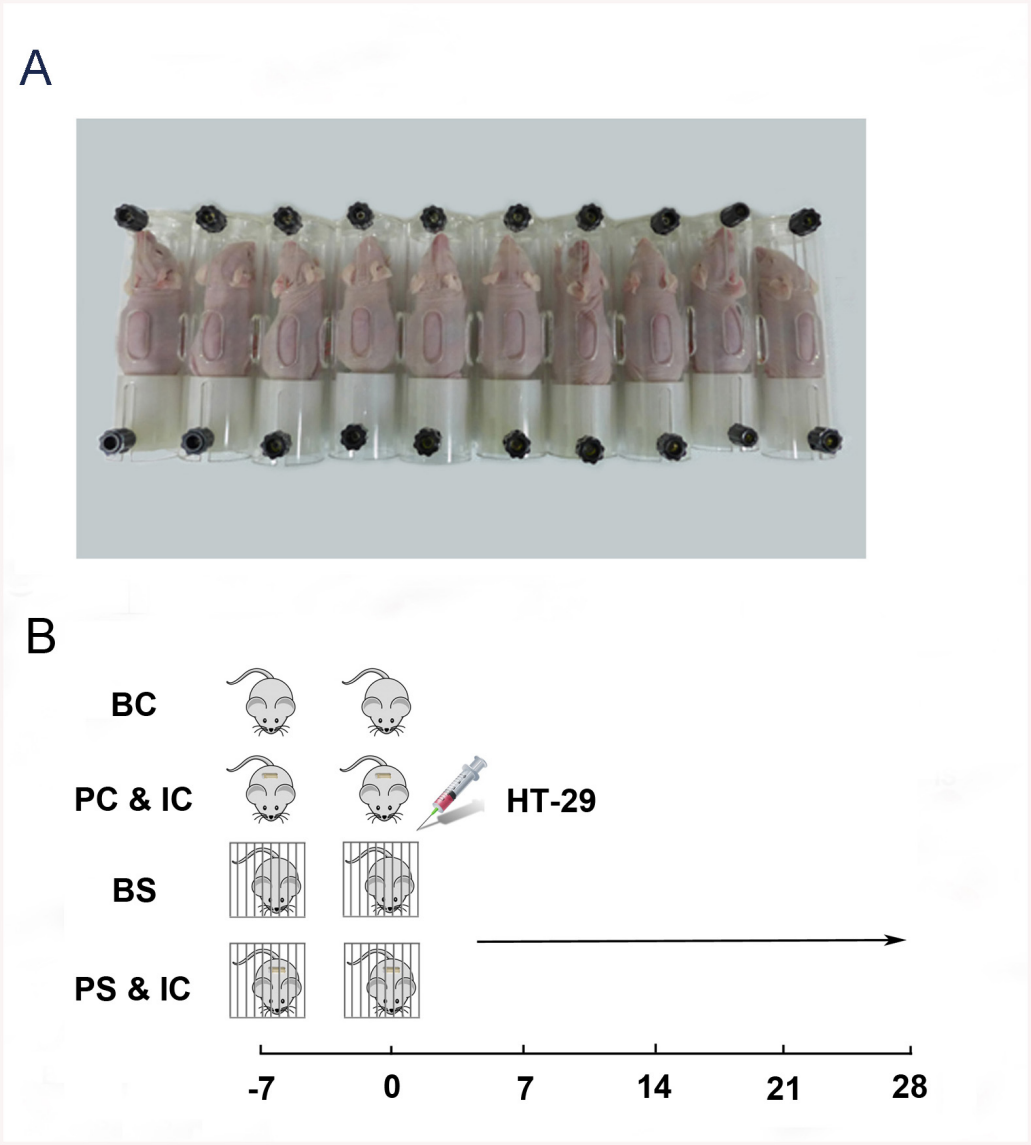
Chronic restraint stress and drug administration protocol. (A) Mice in the stress groups were restrained in a special space that prevented them from moving freely. (B) Mice in the stress groups experienced physical restraint for 6 hours per day for 35 days commencing 7 days before tumor cell injection. A β-adrenergic antagonist was given subcutaneously by osmotic minipump. Drug exposure was sustained throughout the experiment. HT–29 cells were injected into the spleen on day 0.

For β-adrenergic antagonist studies, (±)-propranolol hydrochloride (a nonselective β-blocker, 10 mg/kg/day, Sigma, P0884) and ICI118,551 (a selective β_2_-blocker, 25μM/30μl, Sigma, I127) [17] were delivered to mice subcutaneously by osmotic minipump (Alzet, Model 1004). Drugs were given for the duration of the experiment commencing 7 days prior to tumor cell injection (Fig 1).

### Enzyme-linked immunosorbent assay (ELISA)

Twenty-eight days after tumor cell injection, mice were sacrificed and blood was harvested from the eyes and immediately placed in clean heparin-coated Eppendorf tubes. E and NE concentrations in the serum were measured by ELISA (R&D Systems) according to the manufacturer’s instructions.

### High-performance liquid chromatography with electrochemical detection

The levels of catecholamines in tumor tissue were quantified using a high-performance liquid chromatography column equipped with an electrochemical detector. The tumor tissues were washed in phosphate buffered saline to remove remaining blood. Samples were then sonicated in cold 1 mol/L perchloric acid. The centrifuged supernatants were collected and filtered with a 0.22 μm filter plate (Millipore). The HPLC-ECD system comprised a reverse-phase column (Agilent 1100, 4.6mm×12.5mm, USA), and an electrochemical detector (Couloshem III, ESA, USA). The mobile phase contained 5% methanol in deionized water, 85 mmol/L citric acid, 300 mmol/L sodium citrate, 0.2 mmol/L EDTA, 1.2 mmol/L sodium octane sulfonate, and distilled water. The flow rate was 1 ml/min, and the pH was adjusted to 4.0 with 2 mol/L sodium potassium.

### Flow cytometric analysis

To analyze infiltration of tumor associated macrophages (TAMs) and myeloid derived suppressed cells (MDSCs) infiltration by flow cytometry, single-cell suspensions were prepared from tumors implanted in the spleen harvested 28 days after injection and incubated with antibodies directly conjugated to fluorochrome labels as previously described [18]. To obtain single cell suspensions, the spleen was dissected and homogenized in Dulbecco’s modified eagle medium (Invitrogen), supplemented with 10% FBS, 100 U/ml penicillin, and 100 μg/ml streptomycin. The erythrocytes were eliminated by incubating with a hemolytic solution (155 mmol/L NH4Cl, 10 mmol/L KHCO3, pH 7.2) for 5 min. To analyze TAMs, splenocytes (1 × 10^6^) were stained with PE anti-mouse F4/80 (BioLegand, 123110) and FITC anti-mouse/human CD11b (BioLegand, 101206). To analyze monocytic myeloid-derived suppressor cells (M-MDSC) and granulocytic myeloid-derived suppressor cells (G-MDSC), splenocytes were stained with Mouse MDSC Flow Cocktail2 with Isotype Ctrl (BioLegand, 147003). Antibodies were diluted 1:100 in flow cytometry buffer (phosphate-buffered saline with 5% fetal bovine serum). All analysis was conducted on BD LSRII flow cytometer (Beckman Coulter).

### Quantitative real-time reverse transcription-PCR (RT-PCR)

RT-PCR was used to quantify the expression of metastasis-related genes including IL–6, VEGF, MMP–9, TGF-β and PTGS2. Total RNA was extracted from tumor tissue in the spleen with TRIZOL reagent (Invitrogen), and the concentration and purity were determined by a UV spectrophotometer. Then, 1μg RNA was subjected to reverse transcription into cDNA with PrimeScript RT™ reagent kit (Takara, Dalian, China). Real-time quantitative PCR was performed using SYBR^®^ Premix Ex Taq™ II (Takara, Dalian, China). The reaction conditions were: pre-denaturation at 95°C for 30 s and a total of 40 cycles of denaturation at 95°C for 5 s, annealing at 60°C for 34 s and extension at 72°C for 30s. Samples were analyzed in triplicate and expression was normalized to GAPDH expression. The amplification was performed using the following primers: GAPDH sense (5’-AGGTCGGTGTGAACGGATTTG–3’) and antisense (5’-TGTAGACCATGTAGTTGAGGTCA–3’), IL–6 sense (5’-TAGTCCTTCCTACCCCAATTTCC–3’) and antisense (5’-TTGGTCCTTAGCCACTCCTTC–3’), VEGF sense (5’-GCCAGACAGGGTTGCCATAC–3’) and antisense (5’-GGAGTGGGATGGATGATGTCAG–3’), MMP9 sense (5’-CTGGACAGCCAGACACTAAAG–3’) and antisense (5’-CTCGCGGCAAGTCTTCAGAG–3’), TGF-β sense (5’-AGACCACATCAGCATTGAGTG–3’) and antisense (5’-GGTGGCAACGAATGTAGCTGT–3’), PTGS2 sense (5’-TGAGCAACTATTCCAAACCAGC–3’) and antisense (5’-GCACGTAGTCTTCGATCACTATC–3’). Primers were synthesized by Shanghai Sangon Biotech.

### Western blotting

Total protein was extracted from tumor tissue in the spleen as described previously [15], and protein concentration was determined by BCA protein assay kit (Beyotime, P0012). Western blotting was performed with the following primary antibodies: anti-IL–6 (1:500, Abcam, ab154367), anti-VEGF (1:1000, Abcam, ab46154), anti-MMP–9 (1:500, Abcam, ab38898), anti-TGF-β(1:1000, Abcam, ab31013), andanti-PTGS2 (1:500, Abcam, ab62331), followed by incubation with anti-rabbit horseradish-peroxidase-conjugated secondary antibody (1:1000). Proteins were visualized by an ECL chemiluminescence detection system. Detection of GAPDH (1:2000, Abcam, ab181602) served as internal loading control.

### Immunohistochemistry

Specimens from liver metastasis were fixed in 10% buffered formalin and processed for histological examinations. The sections were incubated overnight at 4°C with the following primary antibodies: anti-VEGF (1:200, Abcam, ab1316), anti-CD31 (1:200, Abcam, ab28364), or anti-MMP–9(1:200, Abcam, ab38898). The slides were rinsed and incubated in biotinylated secondary antibody (1:200). Intensity of staining for VEGF, CD31, and MMP–9 expression was measured by Image-Pro 6.0. The mean of 3 integrated optical density values was considered as the expression intensity of protein in this field.

### Statistics

Statistical analyses were performed using SPSS 18.0 (SPSS Inc.). Results were expressed as the mean ± SEM for all paired statistical comparisons. Statistical analysis was performed by a Student's *t*-test. A value of *P*< 0.05 was considered significant.

## Results

### Chronic stress-induced β-adrenergic receptor activation mediated liver metastasis

**Fig 2:**
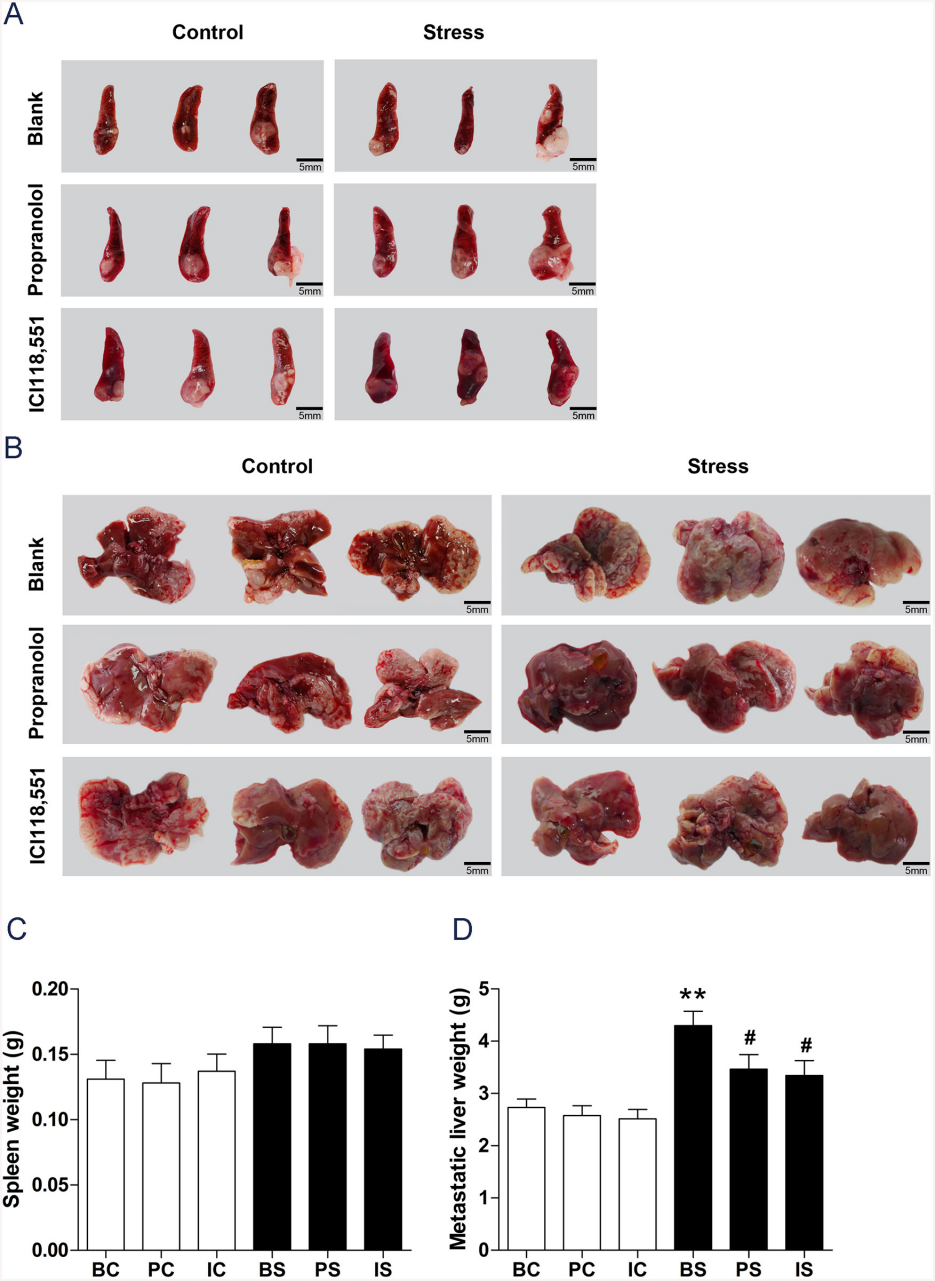
Effect of chronic stress on liver metastasis. (A) Representative images of spleen taken on day 28 after tumor cell injection. (B) Representative images of metastatic liver taken on day 28 after tumor cell injection. (C) Data represents average spleen weight from the six groups. (D) Data represents average liver weight from the six groups. ** *P*< 0.01 versus BC group, ^#^ *P*< 0.05 versus BS group.

HT–29 cells (1×10^7^) were injected into the spleen of 60 mice to establish liver metastasis and sacrificed 28 days after tumor cell injection for analysis. All the mice that underwent tumor injection in the spleen developed liver metastasis. Representative images of spleen and metastatic liver are shown in Fig 2A and 2B. To investigate the role of chronic stress on primary tumor growth and liver metastasis, we evaluated the weights of spleen and liver. Chronic stress increased the spleen weight by 20.61% (BC *vs*. BS: 0.13 ± 0.01g *vs*. 0.15 ± 0.01g, *P* = 0.1784), and a significant increase in liver metastasis was observed in mice exposed to chronic stress (1.6-fold change: 2.73 ± 0.16 g in BC group *vs*. 4.30 ± 0.27 g in BS group, *P* < 0.0001, Fig 2C). These findings suggest chronic restraint stress promotes liver metastasis.

As previous studies suggested that chronic stress accelerated cancer progression through β-adrenergic signaling, we treated mice with propranolol or ICI118,551 to inhibit β-adrenergic signaling. As shown in Fig 2D, β-blocker treatment reduced liver metastasis induced by chronic stress. Compared to mice in the BS group, mice in the PS and IS groups had fewer liver metastases. The metastatic liver weights of mice in the PS and IS groups were significantly lower than those of mice in the BS group (PS *vs*. BS: 3.46 ± 0.28 g *vs*. 4.30 ± 0.27 g, *P* = 0.0482; IS *vs*. BS: 3.35 ± 0.28 g *vs*. 4.30 ± 0.27 g, *P* = 0.0265). We observed no significant difference in liver metastasis between propranolol and ICI118,551 treatment (*P*> 0.05).

### Chronic stress up-regulates catecholamine levels

**Fig 3:**
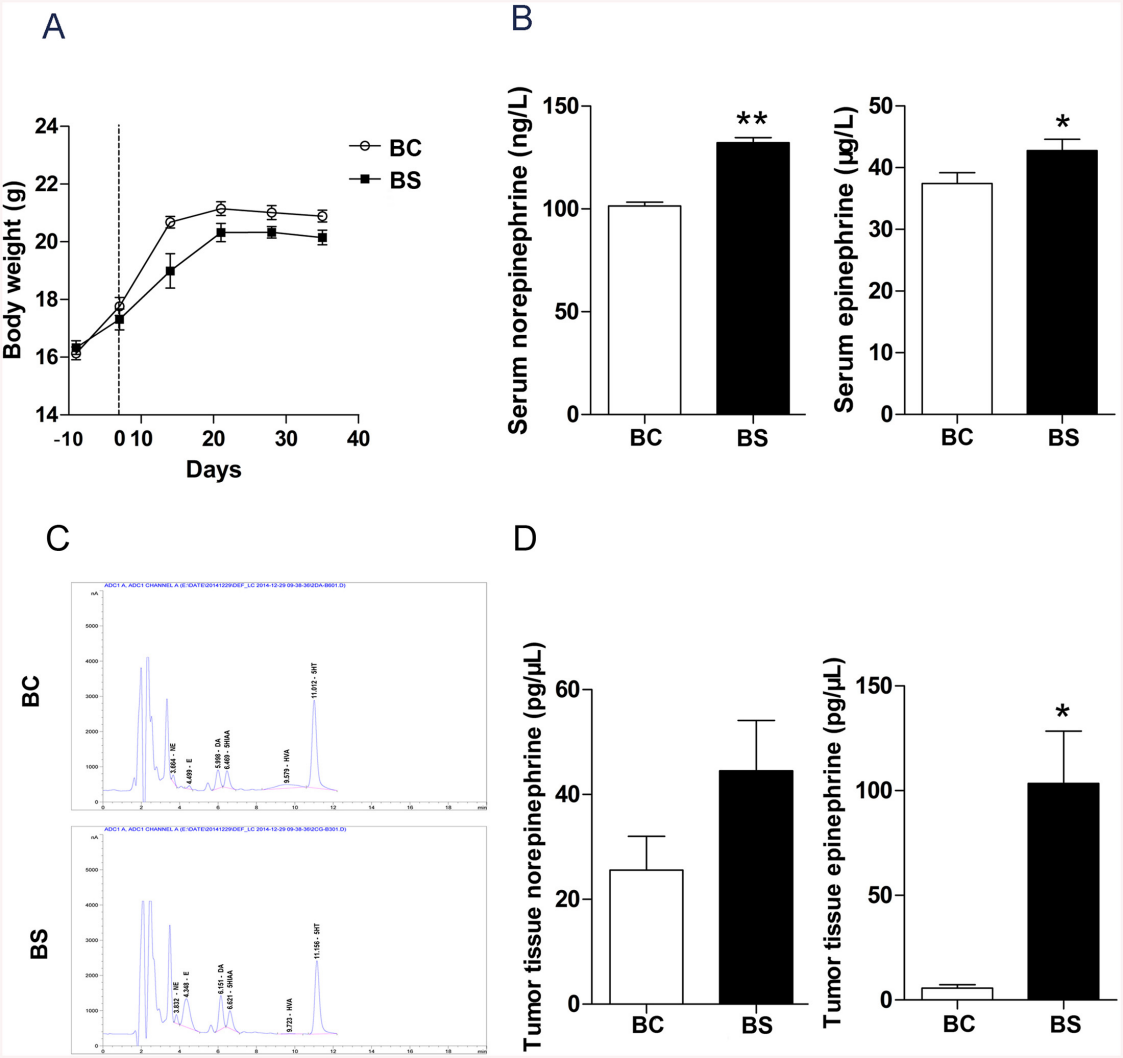
Effect of chronic stress on catecholamine levels. (A) Body weights were monitored throughout the experiment. HT–29 cells were injected into the spleen on day 0. (B) Data represent average serum levels of NE and E in BC and BS group, * *P*< 0.05 versus BC group, ** *P*< 0.01 versus BC group. (C) Representative images of HPLD-ECD fingerprint of catecholamine levels in spleen tumor tissues in BC and BS group. (D) Data represent average level of NE and E in spleen tumor tissue in BC and BS group, * *P*< 0.05 versus BC group.

A central feature of the chronic stress response is up-regulation of the adrenergic stress response pathway, which results in weight loss and increased catecholamine levels [19]. Psychological stress was confirmed by weight lose (Fig 3A) and exposure to chronic stress significantly increased serum levels of NE by 30.25% (BC *vs*. BS: 101.5 ± 1.78 ng/L *vs*. 132.2 ± 2.51 ng/L, *P* < 0.0001, Fig 3B), and moderately increased serum levels of E by 14.24% (BC *vs*. BS: 37.43 ± 1.76 μg/L *vs*. 42.76 ± 1.82 μg/L, *P* = 0.047, Fig 3B). HPLC-ECD analysis of spleen tumor tissue showed significant increase of E by 18.3-fold (BC *vs*. BS: 5.657 ± 1.613 pg/μL *vs*. 103.4 ± 25.08 pg/μL, *P* = 0.0177, Fig 3C and 3D).

### Effect of β-adrenergic signaling pathway on tumor microenvironment

Previous research indicated that β-adrenergic signaling regulates the tumor microenvironment by recruiting or modifying the activity of tumor-associated macrophages [20]. To determine whether chronic stress affects the tumor microenvironment, we used flow cytometry to quantify the cell composition in HT–29 primary tumors harvested from the spleen.

**Fig 4:**
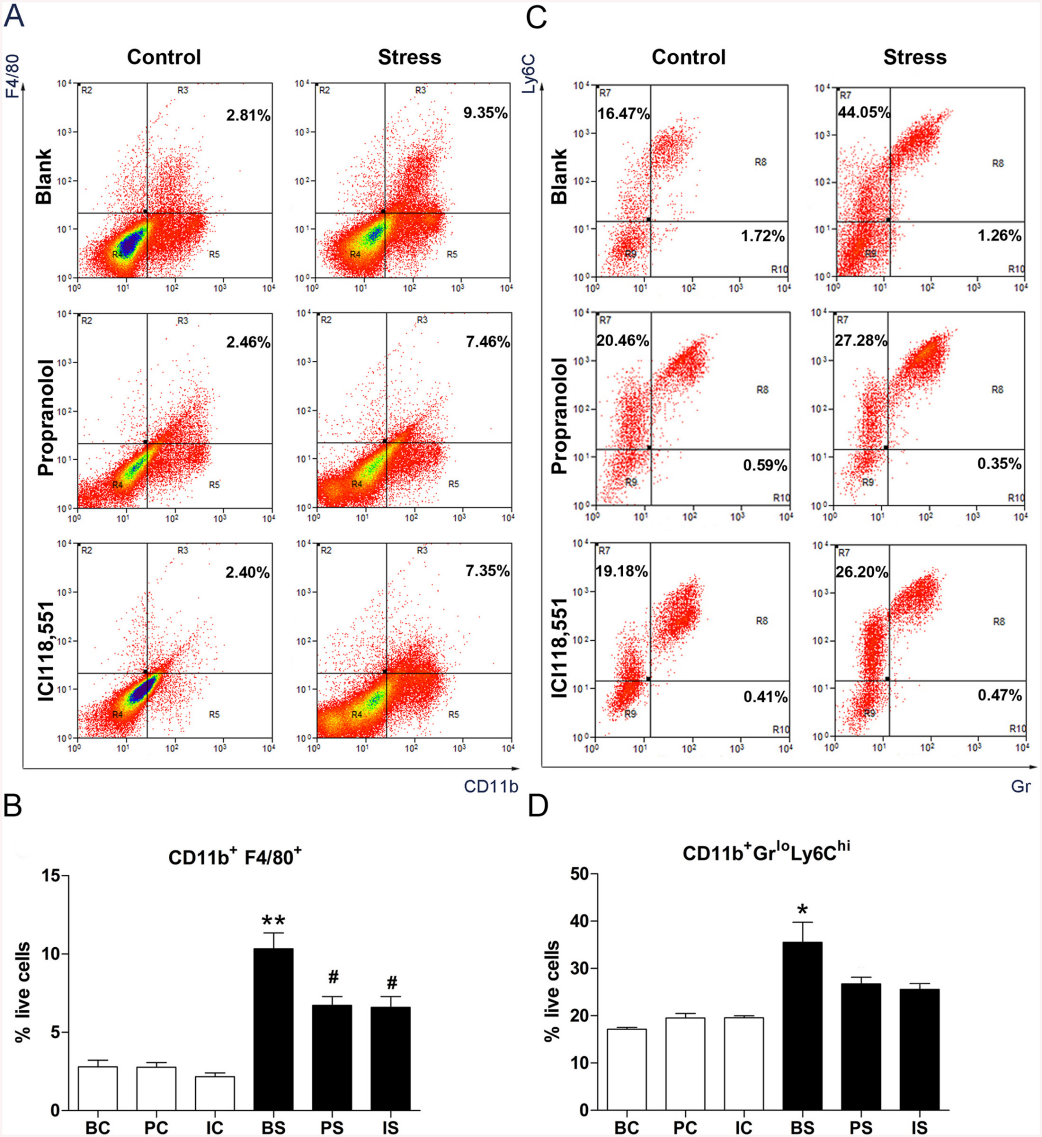
Effect of chronic stress the on the tumor microenvironment. (A) CD11b^+^F4/80^+^ cells were quantified by flow cytometry in HT–29 primary spleen tumors harvested 28 d after injection. (B) Data represents average CD11b^+^F4/80^+^ composition in tumors harvested from the spleen. ** *P*< 0.01 versus BC group, ^#^ *P* < 0.05 versus BS group. (C) MDSCs were quantified by flow cytometry in HT–29 primary spleen tumors harvested 28 d after injection. (D) Data represents average CD11b^+^Gr^lo^Ly6C^hi^ composition in tumors harvested from the spleen. *P < 0.05 versus BC group.

As shown in Fig 4A, chronic stress increased CD11b^+^F4/80^+^ macrophages infiltration (4.2-fold change: 2.79 ± 0.43% of live cells in BC group *vs*. 10.33 ± 1.01% in BS group, *P* = 0.0023, Fig 4B). Chronic stress also increased the M-MDSCs infiltration by 2.4-fold (BC *vs*. BS: 17.15 ± 0.38% of live cells *vs*. 35.51 ± 4.27%, *P* = 0.0128, Fig 4C and 4D). Chronic stress primarily-induced recruitment of tumor associated macrophages and M-MDSCs to the primary tumor, as G-MDSCs were not present (*P* > 0.05). To confirm that tumor associated macrophages and M-MDSCs infiltration were mediated by the β-adrenergic signaling pathway, we analyzed the cell composition of spleen tumor tissue harvested from stressed mice treated with propranolol or ICI118,551. As shown in Fig 4, β-blocker treatment significantly abrogated the CD11b^+^F4/80^+^ macrophages population in the primary tumor tissue (PS *vs*. BS: 6.71 ± 0.57% of live cells *vs*. 10.33 ± 1.01%, *P* = 0.0353; IS *vs*.BS: 6.60 ± 0.68% of live cells *vs*. 10.33 ± 1.01%, *P* = 0.0374). However, no significance was observed in the CD11b^+^Gr^lo^Ly6C^hi^ MDSC populations (PS *vs*. BS: 28.08 ± 0.80% of live cells *vs*. 35.51 ± 4.27%, *P* = 0.2722; IS *vs*.BS: 26.77 ± 0.57% of live cells *vs*. 35.51 ± 4.27%, *P* = 0.2116).

### Effect of β-adrenergic signaling pathway on liver metastasis

**Fig 5:**
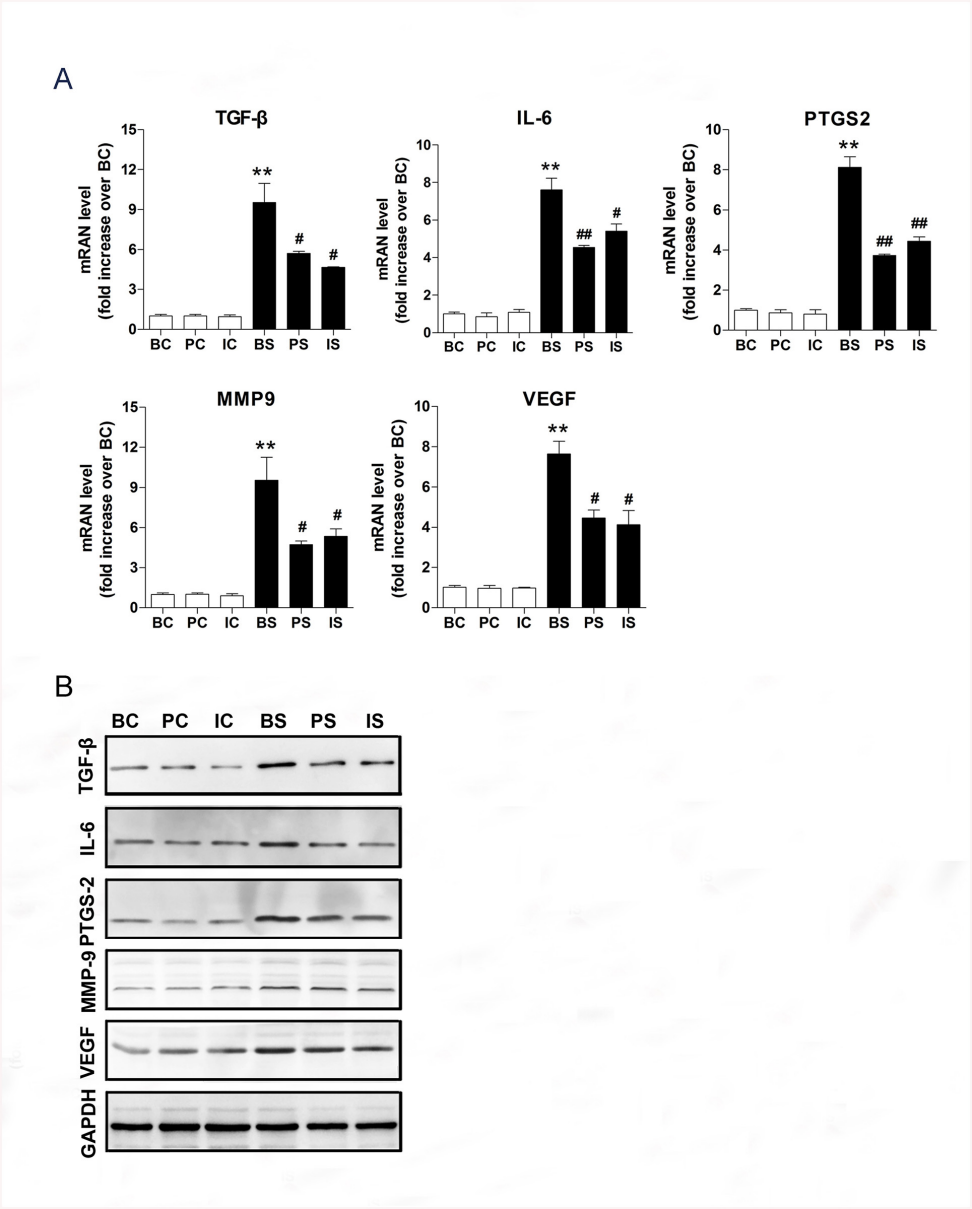
Effect of β-adrenergic signaling pathway on metastatic genes. (A) mRNA expression of TGF-β, IL–6, PTGS2, MMP–9, and VEGF relative to mice in BC group in spleen tumors.** *P*< 0.01 versus BC group, ^#^ *P* < 0.05 versus BS group, ^##^ *P* < 0.01 versus BS group. (B) Protein expression of TGF-β, IL–6, PTGS2, MMP–9, and VEGF in spleen tumors. GAPDH was used as a loading control.

Previous studies suggest that chronic stress alters the tumor microenvironment and may contribute to cancer metastasis *in vivo* [21]. To determine whether chronic stress promoted liver metastasis, we examined expression of TGF-β, IL–6, PTGS2, MMP–9, and VEGF in spleen tumors by RT-PCR and Western blot analysis. We found that TGF-β (9.4-fold increase, *P* = 0.0040), IL–6 (7.6-fold increase, *P* = 0.0005), PTGS2 (8.1-fold increase, *P* = 0.0002), MMP–9 (9.4-fold increase, *P* = 0.0081), and VEGF (7.6-fold increase, *P* = 0.0005) mRNA expression significantly increased in mice in the BC group as compared to mice in the BS group. Mice treated with β-blockers had significantly decreased TGF-β, IL–6, PTGS2, MMP–9, and VEGF mRNA expressions in spleen tumors compared to mice in the BS group (Fig 5A). No significant differences were observed between propranolol and ICI118,551 treatments (*P* > 0.05).

We also observed increased protein expression of TGF-β, IL–6, PTGS2, MMP–9, and VEGF protein in mice exposed to chronic stress, which was abrogated by treatment with β-blockers (Fig 5B).

**Fig 6:**
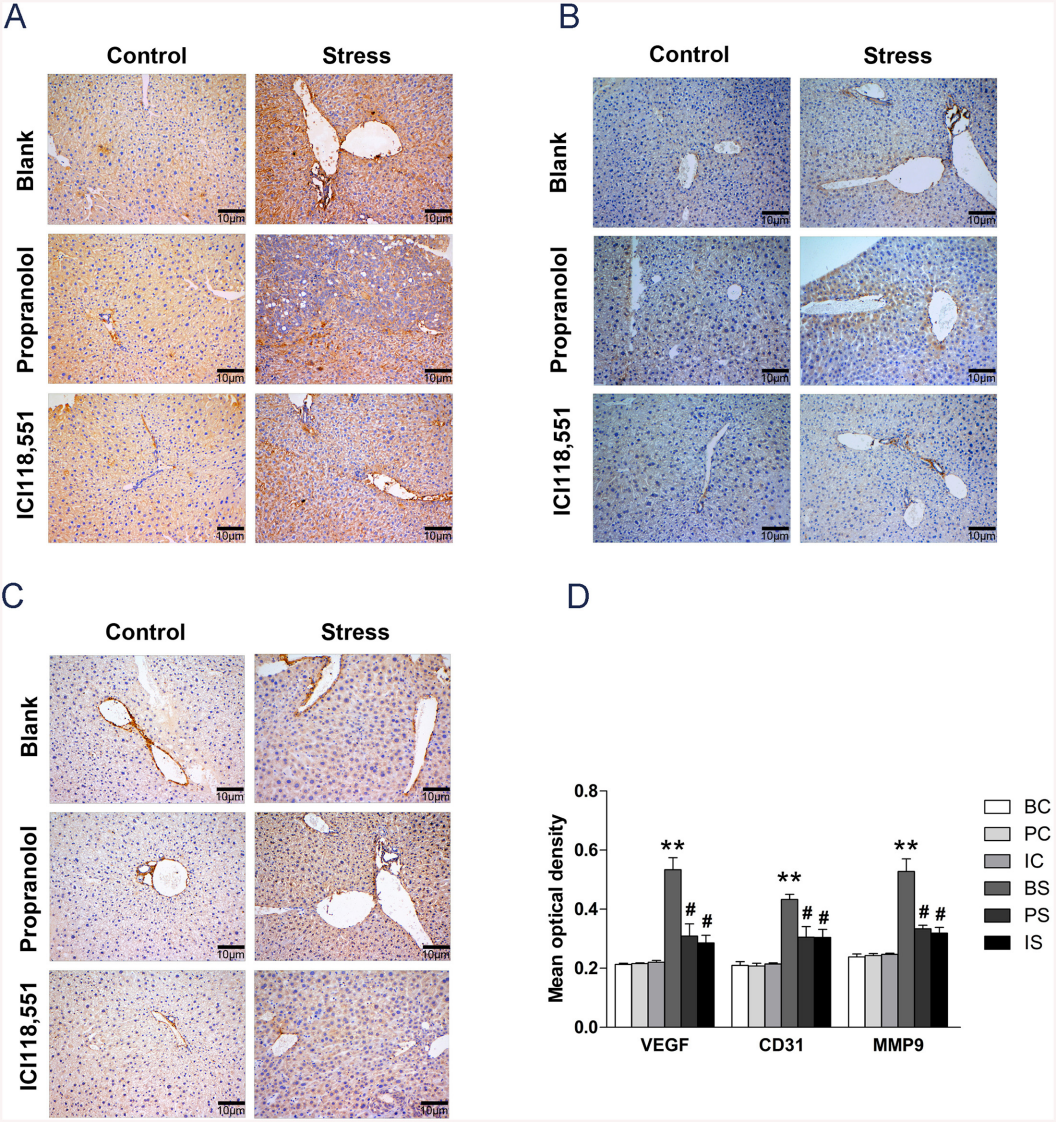
Effect of β-adrenergic signaling pathway on tumor vascularization and tissue invasion. (A)Liver metastatic tumor samples were stained for VEGF by immunohistochemistry. (B) Liver metastatic tumor samples were stained for CD31 by immunohistochemistry. (C) Liver metastatic tumor samples were stained for MMP9 by immunohistochemistry. All photographs were taken at 200×. (D) Mean optical density (OD) of VEGF, CD31, and MMP9 in metastatic liver tumors from mice in the BC, PC, IC, BS, PS, and IS groups. ** *P*< 0.01 versus BC group, ^#^ *P* < 0.05 versus BS group, ^##^ *P* < 0.01 versus BS group.

Previous studies suggest that chronic psychological stress promotes metastasis by up-regulation of tumor neovascularization and tissue invasion. Here we show that VEGF, CD31, and MMP9 protein expression increases in liver tissue indicative of angiogenesis and invasion. Expression was significantly increased in mice in the BS group compared to mice in the BC group (VEGF: 2.5-fold increase, *P* = 0.0014; CD31: 2.1-fold increase, *P* = 0.0004; MMP9: 2.2-fold increase, *P* = 0.0028). Inhibition of β–adrenergic signaling decreased VEGF (PS *vs*. BS: 0.3091 ± 0.04158 *vs*. 0.5340 ± 0.04043, *P* = 0.0179; IS *vs*. BS: 0.2854 ± 0.02639 *vs*. 0.5340 ± 0.04043, *P* = 0.0067), CD31 (PS *vs*. BS: 0.3053 ± 0.03593 *vs*. 0.4333 ± 0.01677, *P* = 0.0320; IS *vs*. BS: 0.3040 ± 0.02751 *vs*. 0.4333 ± 0.01677, *P* = 0.0160) and MMP9 (PS *vs*. BS: 0.3338 ± 0.01203 *vs*. 0.5277 ± 0.04289, *P* = 0.0121; IS *vs*. BS: 0.3187 ± 0.01987 *vs*. 0.5277 ± 0.04289, *P* = 0.0115) protein expression (Fig 6). Together, our results suggest that activation of β-adrenergic signaling is necessary and sufficient to enhance tumor angiogenesis, invasion, and metastasis in the presence of chronic stress.

Collectively, our findings suggest that chronic stress promotes CRC liver metastasis through β-adrenergic signaling, and emphasizes the importance of the tumor microenvironment in regulating tumor cell invasion.

## Discussion

**Fig 7:**
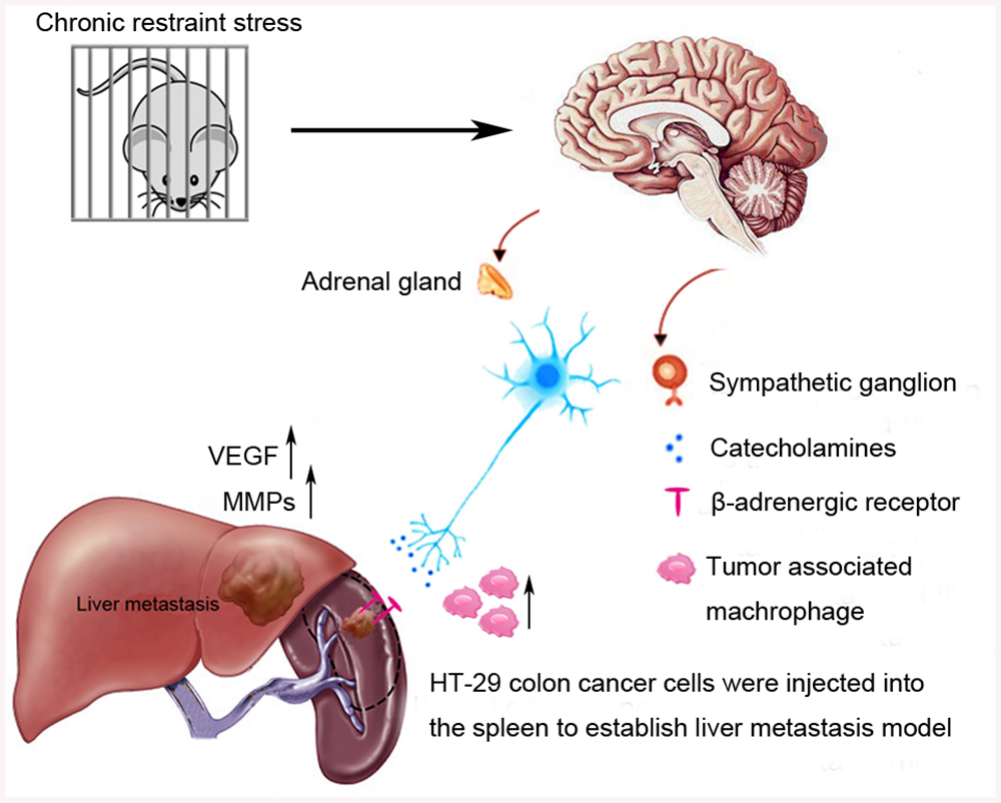
Effect of chronic psychological stress on liver metastasis of colon cancer in mice. β-adrenergic signaling significantly enhanced tumor cell dissemination to liver, and pharmacologic inhibition of β-adrenergic signaling reduced liver metastasis. Our studies also suggest that propranolol may complement existing therapeutic strategies to slow or prevent liver metastasis from CRC, improving survival of patients who are suffering psychological stress.

Despite improvements in comprehensive therapy for CRC, clinicians face challenges in treat patients with liver metastasis due to the limited efficacy. In this study, we found that chronic stress increased the growth and metastasis of CRC through β-adrenergic signaling (Fig 7).

The negative impact of chronic stress on the outcome of cancer is well established. Chronic stress activates the SNS and the HPA axis to encourage release of catecholamines, and further regulate the tumor microenvironment and affect tumor progression [19]. Catecholamines are potent stimulators of vascularization. Previous studies show that chronic restraint stress leads to increased levels of tissue catecholamines and enhanced tumor angiogenesis through regulation of VEGF and MMPs expression [22]. Additionally, β-blockers had been shown to reverse the stress-enhanced angiogenesis [23]. Other studies show that chronic stress elevates stress hormones that increase CRC growth *in vivo* [16]. Here, we expand on those studies by investigating the role of catecholamines in CRC metastasis to the liver. Our results demonstrate that chronic stress significantly elevates E and NE levels in serum and tumor tissue.

To determine if chronic stress was sufficient to regulate angiogenesis *in vivo*, we mapped the blood vessel density of liver metastatic tumor through VEGF and CD31 immunostaining and found a significant increase angiogenesis in stressed mice, respectively. Consistent with previous studies demonstrating that SNS regulates angiogenesis, our data suggests β-blocker treatment abrogated stress-induced vascularization. We also show that chronic stress exposure regulates MMP9 expression in liver metastatic tumor. Collectively, our data suggest that β-adrenergic signaling regulates both tumor vascularization and invasion.

Chronic stress may also alter the tumor microenvironment and promote tumor progression by suppressing host immunity [2, 24]. TAMs and MDSCs are two major immunosuppressive cells in the tumor microenvironment. TAMs suppress anti-tumorigenic immune cells, thereby promoting metastasis [25]. However, the mechanism of TAMs involvement in liver metastasis during exposure to chronic psychological stress has not been defined. MDSCs also accumulate in patients reporting high levels of psychological stress [26–27]. Our data confirms previous findings that chronic stress shapes the tumor microenvironment by recruiting more macrophages and MDSCs into primary tumor, which may facilitate metastasis.

Our data show that exposure to chronic stress significantly alters the primary tumor microenvironment, which may facilitate metastasis to distant organs. Importantly, pharmacologic inhibition of β-adrenergic signaling may be a novel strategy for minimizing CRC metastasis to the liver. Our results are consistent with epidemiologic findings linking β-blocker usage to reduced metastasis and colon cancer mortality [28, 29]. Our data raises the intriguing possibility that β-blocker treatment may be a new strategy for the management of chronic stress in patients with CRC. Additionally, our findings highlight the importance of considering patient psychology in the development of new therapeutic approaches to limit cancer progression and minimize metastatic rates in CRC.
